# Alcohol’s Role in HIV Transmission and Disease Progression

**Published:** 2010

**Authors:** Ivona Pandrea, Kyle I. Happel, Angela M. Amedee, Gregory J. Bagby, Steve Nelson

**Keywords:** Alcohol and other drug (AOD) use, abuse, and dependence, AOD effects, disease factor, disease complication, immune system, human immunodeficiency virus (HIV), HIV infection, acquired immune deficiency syndrome, antiretroviral therapy, simian immunodeficiency virus, animal models, rhesus macaques

## Abstract

Alcohol use has negative effects on HIV disease progression through several mechanisms, including transmission, viral replication, host immunity, and treatment efficacy. Research with animal models has explored the effect of alcohol intake on several aspects of simian immunodeficiency virus (SIV) disease progression. Data suggest that the increased SIV levels observed in alcohol-consuming animals may represent an increase in virus production as opposed to a decrease in host defense. Results also suggest that changes in nutritional balance and metabolism, as a possible consequence of a proinflammatory state, together with increased virus production in animals consuming alcohol, accelerate SIV and possibly HIV disease progression. Further studies using the animal model are necessary.

Human immunodeficiency virus (HIV) infection continues to be a major global health problem, with an estimated 33 million people infected as of 2007. In the United States, more than 1.1 million people are infected with HIV (Centers for Disease Control and Prevention 2009). Although effective treatment of HIV infection has substantially decreased HIV-related mortality, HIV still is the sixth leading cause of death for adults aged 25–44 in the United States (http://www.cdc.gov/hiv/topics/surveillance/resources/slides/mortality/slides/mortality.pdf).

Recent data indicate that new HIV cases have not decreased in recent years and may actually be increasing in men who have sex with men, a trend observed both in North America and in Asia (http://www.unaids.org/en/KnowledgeCentre/HIVData/GlobalReport) (Hall et al. 2009).

Alcohol is the most commonly abused substance in the United States, and people with HIV are more likely to use alcohol than the general population. Whereas the rate of alcohol abuse is approximately 4.6 percent in the general U.S. population (Grant et al. 2004), 8 percent of HIV-infected individuals in U.S. cohorts can be classified as heavy drinkers (Galvan et al. 2002). The prevalence of hazardous drinking in HIV-infected U.S. veterans is 20 percent, with 67 percent considered to drink “too much” at some point by their health care providers (Conigliaro et al. 2003). The rate is even greater among homosexual/bisexual men (41 percent) (Lefevre et al. 1995). Heavy drinking in HIV patients correlates with illicit drug use (cocaine and heroin), lower educational status, and male gender (Galvan et al. 2002).

Given the high prevalence of substance abuse in the HIV population, deciphering the effects of alcohol and other drugs on HIV transmission, disease progression, and treatment response is a high research priority. This article will review evidence showing that excessive alcohol consumption is detrimental to the HIV-infected patient in several aspects (see figure 1). Topics discussed include the effect of alcohol abuse on HIV transmission, viral replication, host immune system, and efficacy of treatment to keep HIV levels low (i.e., antiretroviral [ARV] therapy [ART]). The article also will present data showing the effect of alcohol intake on simian immunodeficiency virus (SIV) pathogenesis in rhesus macaques, the animal model of choice for HIV infection. Finally, the article will discuss potential research avenues in nonhuman primate (NHP) animal models that can be used to further our understanding of the role of the alcohol in the natural history of acquired immune deficiency syndrome (AIDS).

## Alcohol’s Role in HIV Transmission

**Figure 2:**
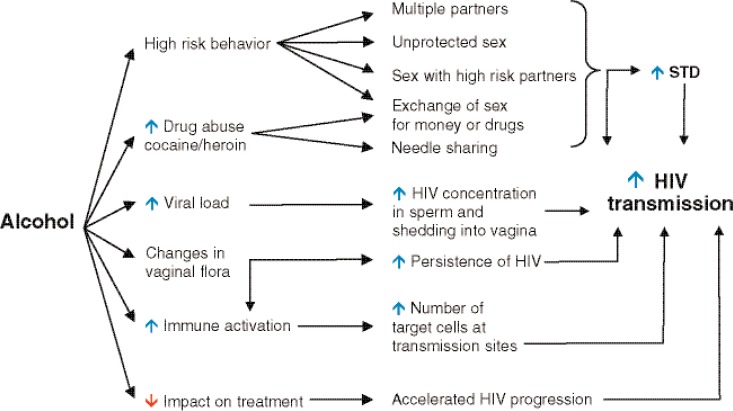
Alcohol consumption may facilitate human immunodeficiency virus (HIV) transmission through risky sexual behavior, increased HIV shedding, and increased inflammation at mucosal sites. NOTES: STD, sexually transmitted disease.

As stated above, alcohol intake increases the rate of HIV transmission through multiple mechanisms, as reviewed below and shown in figure 2.

### Behavioral Effects

Alcohol use causes disinhibition and diminished perception of risk, which increase the likelihood that a person would put him or herself (or his/her partner) at risk for HIV infection by engaging in unsafe sexual practices, such as having multiple sex partners, unprotected intercourse, sex with high-risk partners (e.g., injection drug users, prostitutes), and exchanging sex for money or drugs (Kresina et al. 2002; Windle 1997). However, this issue still is being debated (Raj et al. 2009; Seth et al. 2008; Theall et al. 2007).

Sexual promiscuity triggered by alcohol abuse also increases the risk of acquiring other sexually transmitted diseases (STDs) (Windle 1997), and people with STDs are at risk for both transmitting and acquiring HIV (Wasserheit 1992). STDs appear to increase susceptibility to HIV infection by two mechanisms. Ulcerative STDs such as syphilis, herpes, or chancroid cause breaks in the genital tract lining or skin, creating a portal of entry for HIV. Genital ulcers or nonulcerative STDs such as chlamydiosis, gonorrhea, and trichomoniasis induce inflammation in the genital tract, thus increasing the concentration of cells in genital secretions that can serve as targets for HIV (e.g., CD4^+^ T-cells, a type of white blood cell involved in cell-mediated immunity which also serve as host cells that aid HIV in replication). STDs also appear to increase the risk of an HIV-infected person transmitting the virus, as people with HIV who present with other STDs are shedding more HIV in their genital secretions than are those who are infected with HIV only (Kiviat et al. 1990; Moss et al. 1995). Moreover, the concentration of HIV in semen is significantly higher in patients coinfected with the bacteria responsible for gonorrhea (Plummer et al. 1991).

### Physiological Effects

Aside from behavioral impacts, alcohol abuse may increase host transmissibility through other mechanisms. By increasing viral replication in HIV-infected patients, alcohol may increase the virus concentration in the semen and in the vagina and thus facilitate HIV transmission. Thus, moderate to heavy alcohol consumption is positively correlated with vaginal shedding of HIV in patients on ART, even after adjusting for medication compliance (Theall et al. 2008). By interacting with diverse components of the immune system, alcohol may increase immune activation and inflammation in the HIV-infected patient and thus increase the pool of HIV target cells systemically and at transmission sites. In addition, alcohol may be responsible for changes in vaginal flora, which may induce inflammation and thus increase the rates of HIV transmission (Coleman et al. 2007; Rebbapragada et al. 2008).

Other factors that may contribute to the increased spreading of HIV––such as high levels of viral replication and resistance, increased levels of immune activation, and accelerated disease progression––have been reported to occur in medication noncompliant HIV-infected patients that abuse alcohol.

Altogether, these data clearly suggest that alcohol consumption has a negative effect on HIV transmission and that specific interventions should be developed for substance abuse treatment in the HIV population. These interventions should particularly target women and teenagers, the segment of the population in which most of the new cases arise and in which alcohol plays an important role in HIV transmission.

## Alcohol’s Effects on HIV Pathogenesis

Although earlier studies did not find an association between alcohol consumption and markers of HIV disease progression (Kaslow et al. 1989), recent research has found a significant association between heavy alcohol consumption and lower levels of CD4^+^ T-cells among HIV-infected alcoholic patients not receiving ART (Samet et al. 2007). This effect was not observed among moderate drinkers, however, or among patients adherent to ART. Additionally, alcohol consumption was not associated with higher viral levels in plasma (a blood component).

In women, recent alcohol consumption was significantly associated with the presence of HIV in vaginal secretions (Theall et al. 2008). Plasma viral levels were not associated with recent alcohol consumption, and associations between alcohol use and levels of HIV in the genitals were not observed among women taking ART.

Although these observational studies provide evidence that alcohol use affects viral replication or HIV disease within a host, they also highlight the complexities of study design and of assessing human behavior over a lengthy and variable disease course. To more clearly delineate the associations between alcohol use and HIV disease, investigators have turned to studies using controlled laboratory environments (in vitro) as opposed to studies in living organisms (in vivo).

### Virus Entry and Replication

In a study of blood cells taken from HIV-positive study participants, researchers found that the virus replicates to higher levels in cells taken after alcohol consumption compared with cells obtained before alcohol was consumed (Bagasra et al. 1993). The cells obtained after alcohol ingestion also displayed a reduced capacity for producing interleukin (IL)-2, a molecule that carries signals between cells (i.e., a cytokine) important in immunity. The increased in vitro HIV replication in blood cells from alcohol users also was associated with the inhibition of CD8^+^ T-cells, which attack infected cells (Bagasra et al. 1996).

HIV strains can bind with two specific molecules on the cell surface (i.e., receptors): the chemokine coreceptors CCR5 and CXCR4. Alcohol exposure increases the number of cells with the CXCR4 coreceptor, resulting in enhanced early replication of viral subtypes that use CXCR4 as a coreceptor (Liu et al. 2003). The number of cells expressing the coreceptor CCR5 decreases following alcohol exposure, and, consequently, alcohol treatment of blood cells does not affect the early replication of CCR5-utilizing HIV subtypes, compared with controls. Similarly, in vitro exposure of human skin cells from the mouth to alcohol increases surface expression of CXCR4 and enhances in vitro replication of HIV in these cells (Chen et al. 2004). These in vitro studies identified possible mechanisms by which alcohol may affect the dynamics of HIV infection and replication in the body.

### Alcohol’s Effects on the Immune System

Alcohol consumption affects various components of the immune system, with both chronic and acute alcohol consumption disrupting immune functions (Cook 1998; Goral et al. 2008; Lau et al. 2009; McClain et al. 1993; Molina et al. 2003; Nelson and Kolls 2002; Szabo and Mandrekar 2009). However, little is known regarding the direct interactions between alcohol-induced immunological changes and those induced by HIV infection.

Acute and chronic alcohol consumption have differential effects on the nonspecific (i.e., innate) and acquired (i.e., adaptive) arms of the immune system (Goralet al. 2008).

#### Effects of Acute Alcohol Exposure

Acute alcohol exposure suppresses the production of proinflammatory cytokines, including tumor necrosis factor (TNF)-α and IL-1β both in the cells that ingest foreign material in tissues (i.e., macrophages) in the lungs and in the white blood cells important in immunity (i.e., monocytes) (Goral et al. 2004, 2005, 2008; Imhof et al. 2001; Nelson et al. 1989; Pruett et al. 2004; Szabo et al. 1996*a*, *b*; Verma et al. 1993). Suppression of these immune responses impairs the recruitment of immune cells to sites of infection, and the significance of these immune deficits is well established in the development of bacterial pneumonia and associated disease (Boehm et al. 2003; Kolls et al. 1995; Nelson et al. 2003).

Acute alcohol use also impairs the ability of monocytes and macrophages to bind with molecules that stimulate an immune response (i.e., antigens), thus affecting the efficient production of adaptive immune responses (Mandrekar et al. 2004; Szabo et al. 2004).

#### Effects of Chronic Alcohol Exposure

In contrast, chronic alcohol exposure is associated with increased levels of proinflammatory cytokines, including increased TNF-α production by macrophages (Kishore et al. 2004; McClain et al. 1993). Research with macrophages taken from mice chronically exposed to alcohol shows that these cells have increased levels of the proteins that provide a signal necessary for T-cell activation and survival and that they secrete higher levels of proinflammatory cytokines. Research with mice also has shown that chronic alcohol consumption is associated with more severe lung disease from respiratory syncytial virus (Jerrells et al. 2007). Similarly, chronic alcohol exposure increases the severity of influenza virus–induced disease in the lungs of mice, with increased white blood cell recruitment to the lung, resulting in more severe lung tissue damage and progressive loss of CD8^+^ T-cell function (Meyerholz et al. 2008).

Chronic alcohol consumption, as well as in vitro alcohol exposure, also has been shown to inhibit cytokine production and the functioning of immune cells known as myeloid dendritic cells that process antigen material and present it on the surface to other cells of the immune system (Laso et al. 2007). Recent research (Siggins et al. 2009) has shown that chronic alcohol exposure decreases the number of myeloid dendritic cells in both the bone marrow and circulating throughout the body. Additionally, the expression of proteins important in immune system signaling is suppressed, potentially attenuating antigen presentation and T-cell activation.

Alcohol also inhibits the immune response by decreasing T-cell proliferative responses in vitro and in vivo (Brodie et al. 1994; Chang and Norman 1999; Kaplan 1986), reducing the numbers of CD4^+^ and CD8^+^ T-cells, as well as of immune cells known as natural killer (NK) cells, and altering cell-mediated immunity and cytokine production (Cook 1998; Mandrekar et al. 2004; Meadows et al. 1992; Shellito and Olariu 1998; Song et al. 2002; Starkenburg et al. 2001).

Recent studies in mice on the effects of chronic alcohol exposure on the proliferative responses of both CD4^+^ and CD8^+^ T-cells to specific bacteria antigens showed that chronic alcohol did not affect CD4^+^ T-cell responses but significantly reduced the number of CD8^+^ T-cells responsive to the bacteria and reduced CD8^+^ T-cell proliferation in vivo and in vitro (Gurung et al. 2009).

Increased levels of antibodies have been observed in chronic alcoholics and are associated with increased incidence of autoimmunity. However, the overall number and function of B-cells, which produce antibodies, do not appear to be affected by alcohol (Cook et al. 2007; Paronetto 1993). Collectively, these studies demonstrate that alcohol alters the number and function of immune-cell subsets that play an important role in HIV pathogenesis and thus identify several possible mechanisms through which alcohol consumption may interfere with the natural history of HIV.

## Alcohol’s Effects on ARV Treatment

The development and widespread use of highly active ARV therapy (HAART)^1^, in which several ARV drugs are taken in combination, has substantially reduced morbidity and mortality from AIDS and transformed the lives of many HIV-infected people around the globe (http://www.unaids.org/en/KnowledgeCentre/HIVData/GlobalReport/2008/). However, HAART efficacy is influenced by multiple viral and host factors. The influence of alcohol use on HIV treatment is not yet completely understood and is the subject of intense investigation.

**Figure 3:**
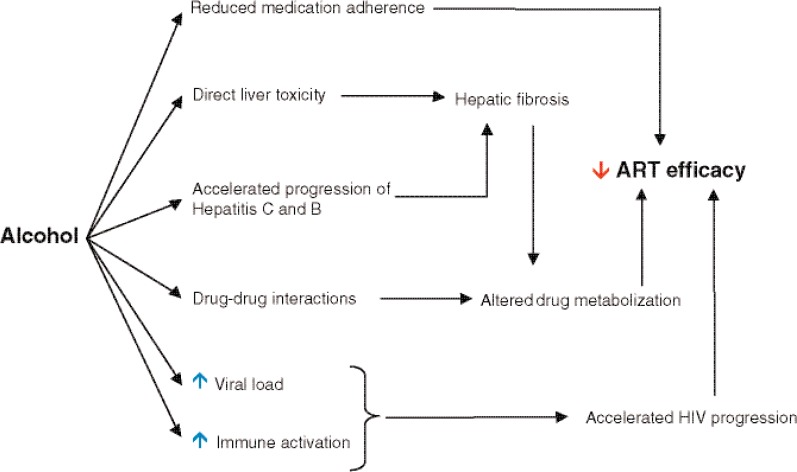
Mechanisms of the alcohol-induced impairment of the efficacy of antiretroviral therapy (ART).

As shown in figure 3, alcohol may effect ART efficacy through diverse mechanisms, including (1) decreasing patient adherence to treatment; (2) altering liver function and ARV drug metabolism; (3) accelerating liver disease in hepatitis C and B, frequent comorbidities in HIV-infected patients; and (4) increasing viral replication or inducing immune activation, which may contribute to a poor HAART response.

### Alcohol and Treatment Compliance

When HAART was first introduced, it became clear that strict compliance to treatment regimens was necessary to achieve undetectable viral loads in HIV-infected patients (Friedland and Williams 1999). Because of the high turnover and mutation rate of HIV and the short half-life of the ARV drugs in use, it is estimated that HIV-infected patients must take 95 percent of their medication in order to avoid the rapid emergence of drug-resistant HIV strains (Kresina et al. 2002).

HIV patient compliance to ART is a function of numerous parameters, including heath insurance, age, socioeconomic status, gender, drug abuse, anger, persistent symptoms, mental problems, and complexity of treatment (Mills et al. 2006). Given the historical association of alcohol abuse with failure to comply with medical treatment, it is not surprising that several studies have demonstrated that alcohol consumption is associated with failure to adhere to prescribed ART (Cook et al. 2001; Lucas et al. 2002; Samet et al. 2004). Drinkers have an almost ninefold increase in medication noncompliance compared with sober patients, and the risk of noncompliance correlates with regimen complexity (Palepu et al. 2003; Parsons et al. 2008). In the backdrop of ART nonadherence by alcohol abusers, it is particularly difficult to directly assess the effect of alcohol on the virologic (and host immune recovery) response to ART (Miguez et al. 2003). Although some studies have shown lower virologic response to ART in drinkers, other studies failed to demonstrate that alcohol modulates ART efficacy (Fabris et al. 2000; Samet et al. 2007). Studies in nonhuman primate (NHP) models of AIDS can address this question by allowing the prospective investigation of alcohol-fed, SIV-infected animals treated continuously or intermittently with ARVs during the entire course of SIV infection (acute and chronic) until progression to AIDS.

### Alcohol and Drug Metabolism

Many ARV drugs undergo significant metabolism in the liver, and there is substantial opportunity for alcohol to disrupt drug bioconversion in HIV patients. Alcohol and other drugs are metabolized by the same enzymes that are involved in the biotransformation of the ARV drugs into toxic intermediates (i.e., by phase 1 enzymes, such as alcohol dehydrogenase, aldehyde dehydrogenase, xantine oxidase, epoxide hydrolase, and cytochrome P450). The toxic compounds are further inactivated by phase 2 enzymes, such as gluthathione *S*-transferase, *N*-acetyl-transferases, sulfotransferases, UDP-glucuronosyltransferases, and methyl-transferase (Kresina et al. 2002). Thus, alcoholic HIV-infected patients treated with ARV drugs are at risk for drug– drug interactions that may either decrease or inappropriately increase the effect of HAART. For example, the plasma concentration of the ARV drug abacavir, which is extensively metabolized by alcohol dehydrogenase, increases up to 41 percent (McDowell et al. 2000). Another potential consequence of drug– drug interactions is increased toxicity and development of liver damage in patients who already are at risk for developing liver disease because of the direct toxicity of alcohol.

The type of alcohol (i.e., ethanol) found in beverages, especially in combination with another type of alcohol (i.e., isopentanols) (which also are found in many alcoholic beverages), induces activity of the enzyme cytochrome P4503A4 CYP3A4 in the liver both in vivo and in vitro (Kostrubsky et al. 1995; Louis et al. 1994). This phase 1 enzyme is responsible for the metabolism and degradation of the majority of the drugs included in HAART. Two types of ARV drugs––nonnucleoside reverse transcriptase inhibitors (NNRTIs), including delavirdine, nevirapine and efavirenz, and protease inhibitors (PIs), such as ritonavir, nelfinavir, lopinavir, saquinavir, and indinavir––are all susceptible to increased liver metabolism as a result of alcohol-mediated induction of the CYP3A4 enzyme system. Increased metabolism of the HAART components will result in subtherapeutic drug levels in HIV patients actively abusing alcohol, and this could then readily lead to suboptimal viral control. This scenario is likely underestimated because the routine monitoring of ARV levels is not common in HIV treatment settings.

Given the finding that variability in CYP3A4 activity already is greater in HIV patients than in uninfected control subjects (even in patients not receiving drugs that alter CYP3A4 activity) (Slain et al. 2000), it is clear that active alcohol abuse in HIV infection is a common and highly relevant factor capable of altering the pharmacokinetics of ARVs.

Chronic alcohol abuse frequently leads to liver dysfunction and loss of liver tissue (i.e., cirrhosis), such that drugs normally metabolized by the liver may induce liver toxicity because of impaired conversion. Two ARV drugs well-known to cause liver toxicity are ritonavir and nevirapine, and practitioners should use caution when prescribing these drugs to alcoholics with liver disease (Martinez et al. 2001; Sulkowski et al. 2000). In the case of PIs, chronic alcohol use induces additive stress in the endoplasmic reticulum (ER), an organelle with known involvement in PI-induced liver toxicity (Ji 2008). All ART agents (particularly PIs) should be used with caution, and liver function should be frequently monitored in HIV patients with alcohol use disorders.

### Effect of Alcohol on Coinfections

An additional risk for alcohol use to complicate HAART is coinfection with viral hepatitis. Approximately 30 percent of HIV patients are coinfected with the hepatitis C virus (HCV), and approximately 10 percent are coinfected with the hepatitis B virus (Soriano et al. 2005, 2007). Heavy alcohol use is an additional risk factor for scarring of the liver (i.e., fibrosis) and cirrhosis in these patients (Benhamou et al. 2001; Di Martino et al. 2001). The incidence of ART liver toxicity is increased in coinfected individuals (den Brinker et al. 2000; Sulkowski et al. 2000, 2002), and alcohol is an independent risk factor for liver injury during ART initiation, especially in HIV patients infected with HCV (Nunez et al. 2001). Also, compared with HIV infection alone, the efficacy of ART in HIV/HCV coinfected patients is impaired in its ability to restore blood CD4^+^ T-cell counts (Greub et al. 2000).

### Other Effects of Interactions Between Alcohol and ARV Drugs

A well-known complication of the ARV drug didanosine in alcoholic HIV-infected patients is inflammation of the pancreas (Whitfield et al. 1997; Yarchoan et al. 1990). This medication therefore should be eliminated from the ART regimens in patients who are actively drinking.

Damage to the parts of the nervous system that carry information throughout the body (i.e., peripheral neuropathy) is another complication of chronic alcohol abuse and, given their neuropathic side effects, limits the use of the NRTIs zalcitabine and stavudine in alcoholics (Moyle and Sadler 1998). Alcohol also increases heart disease risk among patients on ART (Miguez-Burbano et al. 2009).

### Effects of Alcohol on Viral Replication and Host Immune System

Alcohol may increase viral replication, per se, and thus contribute to therapy failure. Also, by activating CD4^+^ T-cells, alcohol may fuel viral replication by increasing the number of target cells and impairing the recovery of CD4^+^ T-cells circulating in the body and cells in mucosal sites (e.g., inside the mouth or nose) after ART, even when controlling for ART compliance (Samet et al. 2003).

Given the central role of HAART in HIV disease management, a more robust understanding of the direct role that alcohol plays in disturbing host and pathogen responses to treatment is clearly needed to better address the needs of these patients. NHP models of AIDS may help define all the facets of alcohol’s effects on ART.

## Animal Models for Studying Alcohol Use and HIV Infection

Although mouse and rat models have been used intensively to investigate alcohol’s effects on innate and adaptive immune responses (Boe et al. 2001, 2003; Brown et al. 2007; Edsen-Moore et al. 2008; Gurung et al. 2009; Heinz and Waltenbaugh 2007; Joshi et al. 2005; Lau et al. 2007; Ma et al. 2006; Meyerholz et al. 2008; Ness et al. 2008; Quinton et al. 2005; Zhang and Meadows 2005; Zhu et al. 2004), such models cannot be used to investigate the role of alcohol in the natural history of HIV. Mice immunodeficiency models do not faithfully reproduce HIV pathogenesis. NHPs, which are genetically close to humans and can be infected with retroviruses related to HIV, may therefore be more valuable models for deciphering the role of alcohol in transmission, pathogenesis, and treatment of retroviruses. This section will present the NHP models currently available for the study of alcohol’s effects on HIV pathogenesis, their characteristics, and their potential use in studies modeling the effect of alcohol in HIV infection.

### NHP Models for Pathogenic SIV Infection

SIV was discovered a few years after the discovery of HIV (Daniel et al. 1985; Letvin et al. 1985). SIV infection in rhesus macaques closely mimics HIV’s pathogenesis, virology, immunology, and pathology in humans, and the progression to AIDS also is similar.

### Research Applications

Over the years, this model has been used for vaccine research (Desrosiers 1990) and to demonstrate some of the key features of HIV infections (Smit-McBride et al. 1998*b*; Veazey et al. 1998). Recently, researchers also have studied virus transmission and dissemination from the mucosal sites of entry in macaques (Keele et al. 2009; Ma et al. 2009). Therapeutic studies in macaques were used to develop several classes of ARV drugs (Van Rompay et al. 1998). Macaques infected with a subtype of SIV, SIVsmm, also served as an animal model for transmission of virus during breastfeeding (Amedee et al. 2004). Finally, macaque studies permit invasive approaches of selective depletion of specific components of the immune system to investigate the correlates of disease progression (Gaufin et al. 2009; Schmitz et al. 1999, 2003).

## SIV Pathogenesis

Similar to HIV infection in humans, SIV in rhesus macaques progresses to AIDS in a variable time frame (Hirsch and Johnson 1994). The hallmarks of pathogenic infection are (1) massive, continuous viral replication (Ho et al. 1995; Perelson et al. 1996; Wei et al. 1995), with the amount of HIV in the blood after stabilizing (i.e., viral load set point) predicting the time of progression to AIDS (Mellors 1998; Mellors et al. 1996, 1997); (2) continuous depletion of CD4^+^ T-cells in the bloodstream (Brenchley et al. 2006; Grossman et al. 2006) that is more pronounced at mucosal sites (Brenchley et al. 2004; Li et al. 2005; Mattapallil et al. 2005; Mehandru et al. 2004; Picker et al. 2004; Veazey et al. 1998); and (3) high levels of T-cell immune activation (Giorgi et al. 1999; Sousa et al. 2002), the magnitude of which has been reported to be predictive of disease progression (Giorgi et al. 1999; Sousa et al. 2002). The interaction among these factors cripples the immune system and eventually results in severe immunodeficiency and death (Brenchley et al. 2006; Brenchley et al. 2006; Grossman et al. 2006; Pandrea et al. 2008).

### Immune Responses

HIV and SIV infections induce immune responses characterized by robust antibody-mediated (i.e., humoral) and cellular immune responses (Brander and Walker 2003; Derdeyn et al. 2004; Richman et al. 2003). However, the role of these responses in controlling viral replication still is unclear. Moreover, continuous immune escape (i.e., virus evasion from specific immune responses) is the hallmark of HIV/SIV infection in pathogenic models (Burns and Desrosiers 1994). Interestingly, during acute SIV/HIV infection, CD4^+^ T-cell levels in the blood show only modest decline. Numerous studies in the SIV macaque model (Li et al. 2005; Mattapallil et al. 2005; Smit-McBride et al. 1998*a*; Veazey et al. 1998) demonstrated that a massive CD4^+^ T-cell depletion first occurs at the mucosal level, where the majority of lymphocytes (i.e., white blood cells, including T-cells) reside. More recent studies (Li et al. 2005; Mattapallil et al. 2005) investigated the mechanism of T-cell destruction during acute infection, including destruction of a subset of CD4^+^ T-cells that recognize and respond to a particular antigen (i.e., CD4^+^ memory T-cells). Together, these reports document a massive destruction of CD4^+^ memory T-cells at multiple tissue sites during acute infection.

Following HIV/SIV infection, heightened immune activation commonly is manifested and associated with increased rates of T-cell destruction, and, as a consequence, increased rates of cell proliferation, increased expression of factors that support programmed cell death (i.e. apoptosis), and disruption of lymphoid and bone marrow architecture resulting in reduced T-cell regenerative capacity (Giorgi et al. 1999; Grossman et al. 2002; Muro-Cachoet al. 1995; Sousa et al. 2002). It recently was suggested that because of compromised mucosal immunity, a greater number of symbiotic and opportunistic bacteria can enter the lymphatic system and blood of the SIV-infected macaques and HIV-infected humans. This theory (known as the microbial translocation hypothesis) is based on recent studies that identified elevated levels of the endotoxin lipopolysaccharide (LPS) in the plasma of HIV-infected patients and SIV-infected macaques (Brenchley et al. 2006). Microbial translocation is the most compelling hypothesis put forth to explain the high levels of immune activation in the pathogenic SIV/HIV infections. This hypothesis centers on the role of a type of receptor that recognizes specific molecules derived from microbes (i.e., toll-like receptors) in the induction of the increased systemic immune activation observed in these progressive infections (Brenchley et al. 2006). Chronic, heightened levels of immune activation during SIV/HIV infection result in the dysfunction of numerous immune cell types (Alter et al. 2005; Braun et al. 1988; Cameron et al. 1994; Chia et al. 1995; Kabelitz and Wesch 2001; Leng et al. 2002; Mariani et al. 2001) that contribute to AIDS progression.

### Advantages

Because of its close similarity to HIV infection, the model of SIV infection of rhesus macaques offers several advantages in identifying the role of alcohol consumption in the natural history of AIDS. These include the ability to (1) control the time and route of infection; (2) monitor and control nutritional and behavioral variables; (3) manipulate and control the timing and quantity of alcohol consumption; (4) conduct longitudinal studies because the duration of infection from its inception to terminal disease with virulent strains of SIV typically is less than 2 years, with greater than 50 percent of the animals euthanized with AIDS within 1 year of inoculation if they are not treated with ARV drugs; and (5) perform studies with and without antiviral therapy. These advantages and the ability to administer alcohol to rhesus macaques makes this model ideal for studying the biomedical impact of alcohol on HIV disease transmission, progression to AIDS, and treatment.

## Research Findings From an SIV Alcohol Model

To study the effect of alcohol on SIV infection in rhesus macaques, the authors developed a model in which intoxicating amounts of alcohol are delivered via a permanently implanted stomach tube (Bagby et al. 2003; Winsauer et al. 2002). This alcohol– rhesus macaque model has been used to study the impact of alcohol on (1) SIV disease progression (Bagby et al. 2006); (2) SIV expression in mucosally inoculated macaques (Poonia et al. 2005); (3) intestinal lymphocyte subsets and turnover before and during SIV infection (Poonia et al. 2006); (4) nutritional, metabolic, and immune alterations during asymptomatic SIV infection (Molina et al. 2006); (5) SIV-associated weight loss (Molina et al. 2008); (6) myeloid dendritic cell function (Siggins et al. 2009); (7) adaptive host defense response to SIV infection; (8) lung SIV expression during pneumococcal pneumonia; (9) neuropsychological deficits during SIV infection (Winsauer et al. 2002); and (10) the development of an ART model. Together, these studies have shown that alcohol has significant impact on SIV infection.

### Alcohol and Disease Progression

**Figure 4:**
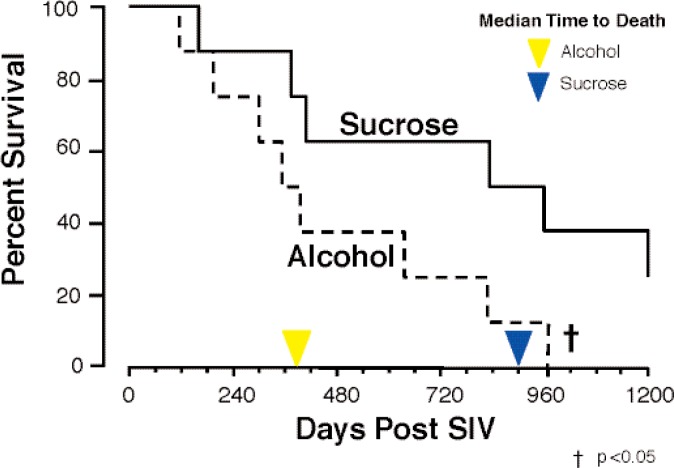
Alcohol administration impacted survival in simian immunodeficiency virus (SIV)-infected rhesus macaques. NOTE: Reproduced with permission from Bagby et al. 2006.

In a major longitudinal study, researchers found that alcohol administration to SIV-infected rhesus macaques resulted in accelerated progression to end-stage disease (see figure 4). Thus, median time of survival decreased from 900 days in sucrose-treated animals to 374 days in alcohol-administered animals (Bagby et al. 2006).

### Alcohol and Viral Replication

**Figure 5:**
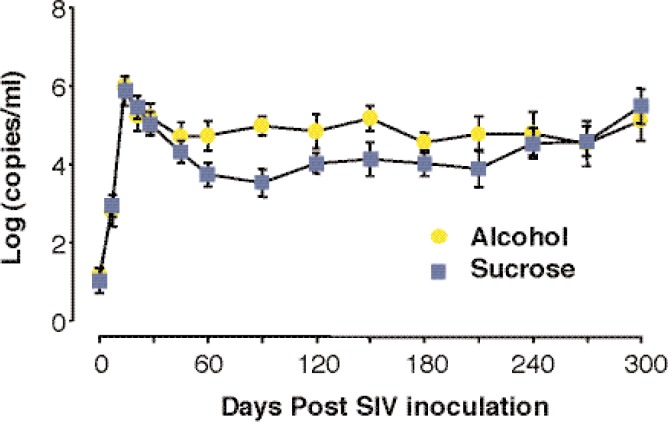
Alcohol administration results in an increase of simian immunodeficiency virus (SIV) replication in rhesus macaques.

In two separate animal studies, researchers reported increased plasma viral loads in alcohol-treated SIV-infected rhesus macaques compared with sucrose-treated animals (Bagby et al. 2006; Poonia et al. 2005). Alcohol administration increases virus levels in plasma by 10- to 100-fold at viral load set point, and SIV was associated with more rapid disease progression in rhesus macaques inoculated either intravenously (see figure 5) or mucosally. Poonia and colleagues (2006) also found consistent increases in viral expression in lymphoid tissue and the intestine in alcohol-treated animals. In a recent study (Poonia et al. 2005), SIV RNA levels were higher in the fluid obtained from the lungs of alcohol-treated animals compared with controls. An additional study by Kumar and colleagues (2005) also demonstrated increased viral replication in the plasma of chronically simian-human immunodeficiency virus (SHIV)/SIV-infected rhesus macaques after a 7-week period of alcohol consumption. The same study reported higher viral loads in the brains of the alcohol-dependent animals compared with control animals. In this study, the increased viral replication was associated with significantly higher CD4^+^ T-cell loss in the alcohol-treated animals (Kumar et al. 2005). Thus, the increased viral turnover in plasma and tissues may explain the accelerated progression to AIDS in the rhesus macaque model.

### Alcohol and T-Cell Subsets

A recent study on subsets of T-cells (Poonia et al. 2006) has shown that the percentage of CD8^+^ T-cells in the small intestine of alcohol-consuming macaques was significantly lower than in sucrose-consuming macaques both before infection as well as in the early postinfection period. Also, the percentage of CD4^+^CD3^+^ lymphocytes in the intestine was significantly higher in alcohol-consuming macaques before infection. To understand the possible reasons behind the increased viral replication, researchers further investigated the effects of chronic alcohol consumption on the percentages of different kinds of lymphocytes in the blood, lymph nodes, and intestine. Although minimal differences were detected in blood and lymph nodes, there were significantly higher percentages of memory T-cells in the intestines from alcohol-receiving animals before infection compared with controls. In addition, higher percentages of antigennaïve T-cells as well as CXCR4^+^CD4^+^ T-cells were detected in intestines of alcohol-treated macaques. Moreover, alcohol consumption resulted in significantly lower percentages of a subtype of memory CD8^+^ T-cell that stimulates other cells (i.e., effector memory cells) as well as activated Ki67^+^CD8^+^ cells in the intestine. Central memory CD4^+^ lymphocytes were significantly depleted in intestines and mesenteric lymph nodes from all alcohol animals at 8 weeks postinfection (Poonia et al. 2006).

These findings suggest that a higher percentage of SIV target cells (memory CD4^+^ T-cells) in the gut, coupled with lower percentages of effector CD8^+^ T-cells (which could be important in controlling virus replication), may be responsible for the higher SIV loads observed in alcohol-consuming macaques (Poonia et al. 2006).

Using a SIV–macaque model of chronic alcohol self-administration, Marcondes and colleagues (2008) also have observed several immunologic changes during the acute phase of infection. These included reduced percentages of circulating memory CD4^+^ T-cells in alcohol-treated animals 7 days after infection, as well as increased levels of CCR5-expressing monocytes 12 days after infection in alcohol-treated animals compared with controls. These data also suggest that alcohol has detrimental effects on the host immune system by increasing depletion of CD4^+^ T-cells and inducing immune activation of other cell subsets that may serve as target cells (Marcondes et al. 2008).

Thus, changes in the circulating and mucosal immune compartments in response to alcohol likely are the major reasons behind higher replication of SIV and rapid disease progression observed in these animals.

Poonia and colleagues (2006) have reported a positive correlation between the number of blood CD4^+^ T-cells and viral load in alcohol-treated animals but not in sucrose-treated control animals. More work is needed, but these observations support the hypothesis that alcohol increases virus production by activating CD4^+^ T-cells.

### SIV, Alcohol, and Body Weight

Bagby and colleagues (2006) reported that body weight loss was more frequently observed in alcohol-fed macaques with end-stage disease than it was in control animals. In other research, Molina and colleagues (2006) observed greater decreases in caloric intake and nitrogen balance in alcohol-receiving monkeys prior to developing AIDS. Although this was not associated with decreased muscle protein synthesis at a relatively early stage of disease, genetic material for the proinflammatory cytokine TNF–α was increased in muscle from alcohol-fed macaques compared with sucrose-treated animals, indicative of a proinflammatory state that might be conducive to later muscle wasting. Such wasting was observed when alcohol-treated animals were followed to end-stage disease (Molina et al. 2008). In this study, alcohol-treated animals with AIDS had significantly lower body weights and limb muscle than did control animals. Research suggests that muscle wasting results from a proinflammatory state, leading to increased muscle weakening via enzymatic breakdown of proteins. These changes in alcohol-fed animals may be in part responsible for their accelerated disease progression during SIV infection.

## Perspectives

Altogether, these results show that alcohol severely affects the natural history of AIDS. Data suggest that the increased SIV levels observed in alcohol-consuming animals may represent an increase in virus production as opposed to a decrease in host defense. Results also suggest that changes in nutritional balance and metabolism as a possible consequence of a proinflammatory state, together with increased virus production in animals consuming alcohol, accelerate SIV and possibly HIV disease progression. More studies using the animal model are necessary in order to determine whether mucosal adaptive immune responses are altered by alcohol.

Mucosal cytotoxic CD8^+^ T-cell responses are important to viral control during SIV infection (Kim et al. 2008; Veazey et al. 2008). The administration of ART to animals early in the course of SIV infection is associated with the development of SIV-specific CD8^+^ T-cells that secrete IL-2, a response that may support gut CD4^+^ T-cell recovery (Verhoeven et al. 2008). In addition, ART is associated with decreased markers of CD8^+^ T-cell proliferation/activation, which may decrease the overall inflammatory milieu of the mucosa. Because ethanol is known to cause mucosal injury/inflammation and decreases mucosal CD8^+^ T-cell populations (Poonia et al. 2006), it is possible that chronic alcohol feeding could detrimentally affect the quality and quantity of cytotoxic mucosal CD8^+^ T-cells during ART.

**Figure 6:**
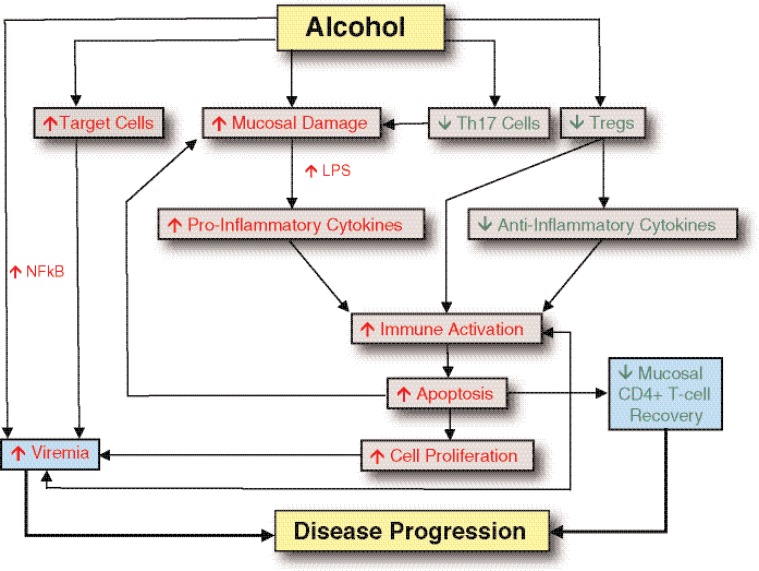
Potential mechanisms of the immune dysfunction induced by alcohol consumption. NOTE: LPS=lipopolysaccharide; NFkB=nuclear factor KB; Tregs=Regulatory T-cells

Alcohol may increase viral replication either by directly increasing the activity of nuclear factor κB, which regulates genes involved in the immune response, or by increasing cell immune activation and thus the pool of target cells (see figure 6).

### Alcohol and Intestinal Inflammation

Chronic ethanol intake is associated with increased intestinal inflammation, decreased expression of proteins that form fluid-impermeable barriers (i.e., tight junction proteins), and impaired barrier integrity (Tang et al. 2008). Research with humans indicates that alcoholics have “leaky” small bowel, and this defect may drive both local and systemic inflammation (Bjarnason et al. 1984). Epigenetic consequences of alcohol abuse may mediate increases in gut permeability. Colonic biopsies from alcoholics (with liver disease) demonstrate increased expression of a microRNA species (i.e., miR-212) that targets transcripts for a specific tight junction protein (Tang et al. 2008). The same study showed that miR-212 expression directly correlates with intestinal epithelial permeability. Alcohol also may contribute to loss of gut integrity by reducing the numbers of intestinal Th17 cells, a T-cell subset reported to play an important role in maintenance of mucosal epithelial integrity (Favre et al. 2009) (see figure 6).

Increased gut permeability also has been described during SIV/HIV infection progression and may play a role in increasing systemic inflammation and virus spread. Thus, the additional alcohol–induced gut injury may directly accelerate progression to AIDS through additional increases in immune activation (see figure 6). Alcohol also may induce immune activation through modifications in the numbers of T–regulatory cells, which inhibit the activation and proliferation of T-cells (see figure 6).

### Alcohol and Immune Activation

By increasing levels of immune activation, alcohol may contribute to increased levels of virus replication and consequently accelerated and more pronounced CD4^+^ T-cell depletion at mucosal sites. Hence, the data obtained using the alcohol–rhesus macaque model clearly demonstrates the adverse effects of ethanol consumption on SIV infection.

NHP studies have shown that ART, when given soon after SIV infection, can protect intestinal memory CD4^+^ T-cells from rapid depletion (Lifson et al. 2003). Moreover, additional studies demonstrated the ability of ART to permit reconstitution of gut CD4^+^ T-cell populations when given 7 days after SIV infection (Verhoeven et al. 2008). However, recent studies of HIV patients who started ART relatively soon after HIV infection suggested that early ART treatment may have a limited impact on recovery of gut memory CD4^+^ T-cells (Mehandru et al. 2006). The disparity between ART studies in HIV-infected patients and SIV-infected NHPs raises the question whether additional factors in human infection, such as alcohol abuse and/or medication compliance, lessen the efficacy of ART. Alcohol abusers are frequently noncompliant with therapy, and this behavior may be at least partially responsible for observations of decreased benefits from ART treatment in this group (Samet et al. 2003; see also the article by Samet in this issue). It also is plausible that increases in viral replication associated with chronic ethanol abuse will increase the number of CD4^+^ T-cells directly destroyed by the virus. Alcohol also may increase CD4^+^ T-cell destruction through increasing immune activation and apoptosis (a mechanism that was reported to be responsible for the death of the majority of CD4^+^ T-cells in HIV-infected humans and SIV-infected macaques). Thus, alcohol may directly interfere with ART capacity to control viral replication and allow CD4^+^ T-cell recovery (see figure 6).

The alcohol–rhesus macaque model is thus ideal to investigate the role of alcohol in modulation of viral replication, mucosal adaptive immune responses, levels of immune activation, levels of apoptosis, numbers of mucosal CD4^+^ T-cells, and ART efficacy because it allows (1) multiple invasive procedures that are necessary to recover gut tissue, (2) sampling at defined time points during the SIV infection, and (3) precisely monitored treatment with ART.

In summary, there is substantial scientific evidence on the adverse consequences of alcohol abuse on HIV infection. The behavioral, virologic, pharmacologic, and immunologic effects of ethanol combine to result in suboptimal treatment responses and the more rapid development of AIDS. Future studies are necessary to decipher the precise mechanisms through which alcohol alters the virus biology and the host immune responses.

## Figures and Tables

**Figure 1:**
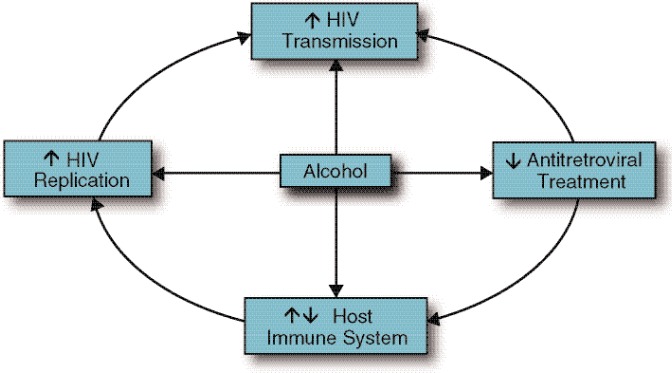
Overall impact of alcohol consumption on human immunodeficiency virus (HIV) pathogenesis.

## Footnotes

1 The acronym HAART now is used interchangeably with the term antiretroviral therapy (ART). Initially, the term HAART was coined to describe the use of a combination of drugs versus a single drug during treatment. Today, all treatments use a combination of drugs; therefore, HAART and ART are equivalent.
